# A qualitative study to inform the design and implementation of AI-driven diagnosis: Challenges, barriers, and clinical insights of physicians

**DOI:** 10.1371/journal.pone.0348519

**Published:** 2026-05-22

**Authors:** Shravya Chitrapady, Pooja Gopal Poojari, Muhammed Rashid, Vijayanarayana Kunhikatta, Muralidhar Varma D, Raviraj V. Acharya, Shivshankar K N, Vasudha Devi, Dinesh Acharya U, Sohil Khan, Girish Thunga

**Affiliations:** 1 Department of Pharmacy Practice, Manipal College of Pharmaceutical Sciences, Manipal Academy of Higher Education, Manipal, Karnataka, India; 2 Department of Pharmacy Practice, Srinivas College of Pharmacy, Valachil, Mangalore, India; 3 Department of Pharmacotherapy, College of Pharmacy, University of Utah, Salt Lake City, Utah, United States of America; 4 Department of Infectious Diseases, Kasturba Medical College, Manipal Academy of Higher Education, Manipal, Karnataka, India; 5 Department of Medicine, Kasturba Medical College, Manipal Academy of Higher Education, Manipal, Karnataka, India; 6 Division of Pharmacology, Department of Basic Medical Sciences, Manipal Academy of Higher Education, Manipal, Karnataka, India; 7 School of Computer Sciences, Manipal Institute of Technology, Manipal Academy of Higher Education, Manipal, Karnataka, India; 8 Pharmacotherapeutics and Evidence Based Practice, School of Pharmacy and Medical Sciences, Griffith University, Gold Coast Campus, Southport, Australia; Kalinga Institute of Medical Sciences, INDIA

## Abstract

**Introduction:**

Acute febrile illnesses are significant clinical challenge in tropical regions due to overlapping symptomatology among infections such as dengue, scrub typhus, and leptospirosis. This diagnostic dilemma leads to delays in appropriate treatment and negatively impacted patient outcome due to limited diagnostic tool availability. Recent advancement in artificial intelligence (AI) application in healthcare help in developing diagnostic aiding tool in such clinical dilemma.

**Aims:**

Study aimed to explore physicians’ perspectives on diagnostic challenges of tropical Acute-febrile-illnesses and assess perceived utility of AI as a supportive tool in clinical decision-making.

**Methods:**

A qualitative interview was conducted using validated semi-structured interview guide using literature and expert opinion and validated by IPR method. To understand the physicians perspective on management infectious diseases in critical care, interview guide administered among ten physicians with 10–30 years of experience across different centres of India. Interviews were audio-recorded, transcribed verbatim, and thematically analyzed using ATLAS.ti software.

**Results:**

Four major themes emerged: clinical experience and practice, diagnostic-challenges, diagnostic-parameters and decision-making, and AI integration in clinical-practice. However, no statistical test can be performed since study mainly focused to understand perception of physicians with respect to managing illness and hence prevalence cannot be determined. Diseases such as dengue, scrub typhus, and leptospirosis were reported as most prevalent, particularly during monsoon seasons. Key diagnostic challenges included overlapping symptoms, diagnostic delays, limited sensitivity/specificity of available tests, and cost constraints. Physicians primarily relied on clinical judgment and laboratory findings, despite the limitations. Most of the participants expressed positive attitudes toward the potential of AI as supportive tool, citing its ability to enhance diagnostic accuracy and streamline clinical workflow with proper validation.

**Conclusions:**

Growing in clinical dilemma AI has significant role in diagnosis of tropical fever with integration of the clinical data & physician perspective can successfully able to incorporate in clinical application.

## Introduction

Acute febrile illness caused by different causatives including bacteria, fungi, parasites etc causing major public health challenges are the significant cause of mortality and morbidity in tropical and sub-tropical regions [1,2]. These diseases might include disease such as dengue, malaria, scrub typhus, leptospirosis, chikungunya, and typhoid etc [3,4]. It contributes around 17% of the global disease burden [5,6]. This may be attributed to many environmental factors like heavy rainfall, poor hygiene, changes in vector habits, under developed drainage facility and water logging places etc. Seasonal variation might influence the major disease outbreaks or the peaks around the year [7,8].

Moreover, most of the physicians confronts with challenges when dealing with these diseases majorly due to its complex diagnostic pathways. Major cause of complicated nature of diagnostic pathway is the overlapping clinical features which includes fever, rash, thrombocytopenia, and jaundice etc and make differential diagnosis difficult [1,2,9]. In the absence of established conventional diagnostics or inadequate access, especially in rural and resource-limited settings, these challenges are further potentiated [1,10]. For this urgent need of improved diagnostic facility, certain scoring systems or decision support systems have been developed which might accurately differentiate between these illnesses at initial presentation. However, there is limitation with respect to its use because of its acceptability and lack of validation in the clinical setting. Furthermore, currently available diagnostics may differ with respect to its accuracy and have high false negative reports. Many available diagnostics are expensive, limiting their practical applicability in the resource limited settings [11].

In the recent years, many fields of healthcare system have been supported by artificial intelligence (AI). Machine learning (ML) based systems are most frequently used technology in any field of medicine. This type of technology serve, being the backbone to the physicians by aiding as clinical decision support systems [12]. However, there is uncertainty in their diagnostic accuracy, clinical relevance and applicability in real world clinical settings. In most of the studies, developed models are based on the clinical symptoms and laboratory investigations, may or may not be supporting differential diagnosis of multiple diseases [7,11]. Few are single centred models and lack external validation, limiting the generalizability of the models. Moreover, the existing studies rarely integrated insights of physicians in development of AI driven diagnostic tools, who are the end users of it [12].

Especially, limited studies captured the clinical challenges faced by physicians in differential diagnosis especially in the emergency setting as well as in resource limited settings. For better understanding on the context and report the exact barrier, problems with respect to diagnosis these diseases, it is essential to examine and understand the existing diagnostic workflow. Moreover, understanding physicians’ views is also necessary for development of AI-based tools, and its applicability. Unlike prior studies that described either diagnostic challenges (clinical and epidemiological aspects of tropical fevers) or development and evaluation of AI models, this study explicitly links physicians experiences and perception with actionable requirements for development and evaluation of AI-based tools.

A recent publication in *The BMJ Open* by the authors, highlights the necessity of understanding physicians perspective on the development of machine-learning based diagnostic tools [7]. The study emphasizes that understanding the physician perception on diagnostic model is essential and have been incorporated for the model development. For this, a qualitative interview have been planned and executed to capture physicians’ insights, expectations, and contextual challenges, thereby ensuring that the resulting tools are both practical and aligned with real-world clinical decision-making.

Hence we conducted a phenomenological study with qualitative approach, to explore physicians’ experiences with diagnosing tropical febrile illnesses and use of AI. The study aims to explore physicians perception with respect to challenges associated with diagnosis of tropical fevers along with their diagnostic decision-making process in order to identify how these insights can be translated to inform development and evaluation of AI based diagnostic tool for differential diagnosis of tropical fevers. Qualitative interview helps in identifying exact need of the physicians and perspective on AI model development for differential diagnosis of tropical fevers. The present study findings are expected to inform crucial aspects of AI model development, such as feature selection for model development, workflow integration, and acceptability of AI in real-world clinical settings.

**Research objectives:**

1. To identify key diagnostic challenges in tropical febrile illnesses.
2. To determine clinical, laboratory, and epidemiological parameters used in decision-making.
3. To explore physicians’ perceptions and expectations regarding AI-based diagnostic tools.

## Methodology

### Study design and participants

A phenomenological qualitative abductive approached study was planned to interpret the lived experience of the physicians who treat tropical infections and febrile illness. We conducted the study to understand the physicians perspective with respect to the importance of clinical, epidemiological and demographical parameters which can be useful in diagnosis. This approach is appropriate as it enables an in-depth understanding of clinicians’ subjective experiences, reasoning processes, and diagnostic challenges beyond what can be captured through quantitative methods.

The institutional ethical approval was obtained before conducting the study. The participants were recruited through purposive sampling and also by snowball sampling [13,14]. The physicians whoever treated and having experience in treatment of tropical fever and febrile illness regardless of their level of experience across Indian clinical setting were recruited. Specifically, physicians working across different level of hospital setting such as secondary, tertiary and quaternary clinical setting practicing in urban, semi urban and rural regions, were included. The physicians who were not willing to give consent were excluded from the study. Potential participants were contacted in advance and they were informed regarding the study. A written or verbal consent was obtained prior to the qualitative interview. Interview was conducted either face to face or online using platforms such as Microsoft Teams or Google meet. Date of first recruitment was 17-Jul-24 and Date of last (latest) recruitment was 3-Apr-25. The study was conducted according to Standards for Reporting Qualitative Research (SRQR) guidelines and checklist provided in the supplementary information S1 Text.

**Ethics Statement:** Institutional Ethics Ethical approval for the study has been obtained from the institutional ethics committee of the Kasturba Medical College and Kasturba Hospital, Manipal (IEC number: **6/2024**). Informed consent has been taken before conducting the qualitative interview and confidentiality of the participants were maintained by anonymizing the interview data prior to the reporting.

### Interview guide preparation & validation

Interview protocol refinement (IPR) framework method was followed during the development of interview guide. IPR involved developing, reviewing, and refining interview questions by obtaining the feedback from the subject experts. The interview guide was developed through the iterative process guided by the literature evidence and consulting with the subject experts. A draft interview guide was then created and validated by the five subject experts using refinement guide provided in IPR framework [15]. Subject experts were asked to assess the content relevance, clarity, comprehensiveness, and appropriateness of the interview guide and feedback was obtained in the form of structured comments and suggestions. We modified the interview guide based on the relevant recommendations provided by the experts during validation. The final interview guide included the questions aligned with the study objectives and was pilot tested with 2 physicians to assess clarity, flow, and timing [15]. Minor modifications were done based on the pilot testing of the interview guide. The developed interview guide covers the domains such as Clinical experience and practice, diagnostic process and Diagnosis & challenges, Parameters in diagnosis, AI in clinical practice.

The first domain focused on the clinical experience and practice of the physician with respect to the tropical fevers which include questions related to commonly encountered diseases, frequency of occurrence and seasonal pattern observed with respect to these diseases.

The second domain, i.e., Diagnosis & challenges were with respect to physicians perspective on diagnostic dilemma, difficulties arising due to overlapping clinical symptoms, challenges encountered despite the availability of laboratory tests and factors affecting diagnostic test accuracy.

The 3rd the main was pertaining to parameters in diagnosis. Here we aim to obtain the opinion on clinical and laboratory variables that might help in development of diagnostic model and to describe how these variables influence the clinical decision making. Additionally potential confounding factor that might affect the diagnosis and that can be considered as parameters in model development were also considered.

4th remain was majorly focused on the application of AI in clinical practice. This domain focused on opinions and experiences regarding desirable features for ML best clinical decision support system, it's expected accuracy levels, perceived challenges in integrating such models into routine clinical practice and the clinical setting that might be most benefited from the implementation of such model.

The domain based summary table describing the conceptual framework and its components have been provided in the Table 1.

**Table 1:** A summary table describing the conceptual framework and its components.

Research Domains	What it studies	Why it matters
Clinical experience	Types & frequency of diseases Physician’s practice and experience on tropical fever management	Provides real-world lived experience of physicians in tropical fever management.
Diagnosis & challenges	Diagnostic difficulties	Research gap in the diagnosis of tropical fevers
Parameters in diagnosis	Clinical & lab variables	Inputs for AI model development
AI in clinical practice	Acceptance & usability	Implementation aspects for success or failure of AI

The validated interview guide have been provided in the supplementary information S2 Text. The Question–Analysis table which helps in mapping the questions in the interview guide with the corresponding analytical approach have been provided in the supplementary information S3 Text.

### Interview process

The validated interview guide was used to conduct the indepth semi structured face to face or online based interviews with the participating physicians. The interviews were conducted by a PhD student who had received formal training in qualitative interview methods. Potential participants were contacted in advance, and they were informed regarding the purpose and procedure of the study. We also informed regarding data confidentiality, and voluntary consent to the study. Prior to the interview, informed consent was obtained verbally or in writing, and the interview was audio recorded. Verbal consent was documented through audio recording, while written consent was recorded in script form. Participants were asked about their clinical experience in diagnosis and challenges, relevant diagnostic parameters and their perspective with respect to the use of AI in clinical practice. Each interview lasted approximately around 20–30 minutes. We continued the interview until we reached data saturation. Currently, during this study process, saturation was reached after eight interviews, and two additional interviews were conducted to confirm stability of the themes. Therefore, a total of ten participants were included.

### Data collection and analysis

After the face-to-face interview with the physicians, the recorded audio was transcribed verbatim. Each transcript were read multiple times to ensure accuracy and data familiarization to capture all the context of the participants experience. Reflexive thematic analysis by Braun and Clarke’s framework [16] with abductive approach was used. Hence, the involved study approach moves between data-driven insights and existing conceptual understanding in an iterative manner. Initially, open coding was performed to identify patterns emerging directly from the data. Subsequently, these codes were examined in relation to existing literature and conceptual understanding of clinical reasoning and diagnostic processes. Data collection and analysis were conducted concurrently, and interviews were continued until no new codes or themes emerged.

Two independent researchers annotated the transcript and the annotated versions were validated by a subject expert. Discrepancies were resolved through the discussion and consensus. The validated transcripts were then coded to obtain the initial coding framework iteratively and updated as new insights emerged. These codes then grouped together to develop categories, followed by developing themes and sub themes. These themes were then validated with subject experts. A total of 2–3 rounds of reviews were conducted for preparing final themes and sub themes. The data management and thematic analysis were conducted using the trial version of ATLAS.ti software [17,18]. In the final themes, sub themes add the respective quotations are provided in the supplementary information S4 Text.

## Results

### Participant characteristics

A total of 10 physicians, specializing in infectious diseases were requested to take part in the qualitative interview, where we conducted one-on-one indepth semi structured interview for 20–30 minutes. Data saturation was achieved during the data collection. Out of 10 participants, 7 participants were recruited through purposive sampling and 3 participants were recruited through snowball sampling. Participants aged between 31–60 years of age with experience of about 12–30 years. The interview was focused on gathering insights regarding the importance of clinical variables, associated epidemiological and demographic factors, and the challenges, dilemmas, and barriers encountered in the diagnostic process. Additionally, participants were asked to share their perspectives on the design and potential usefulness of machine learning models in clinical decision-making.

**Fig 1:**
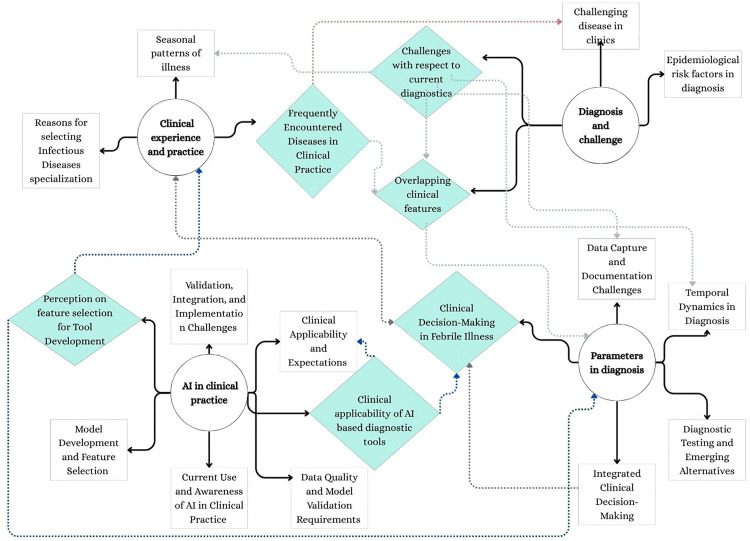
Thematic mapping of the themes and subthemes.

From the thematic analysis, we have obtained 4 themes with 3–5 subthemes per each theme. One focused on clinical experience & practice, 2^nd^ on diagnosis & challenges, 3^rd^ on parameters in diagnosis, and 4^th^ on AI in clinical practice and under every theme we obtained 5–6 subthemes. Thematic mapping of the themes and subthemes have been provided in Fig 1. Throughout these themes, implementation-associated measures such as acceptability, perceived usefulness, potential barriers, and workflow integration of AI-based solutions are embedded and highlighted as appropriate.

**Theme 1: Clinical experience and practice:** Clinical experience of the physicians is one of the important context which guide us to the tropical diseases they are treating, what is their experience in the clinical setting regarding the diagnosis and what are the challenges in their practices. This involves 4 subthemes as follows.

- Sub-theme 1.1: Reasons for selecting Infectious Diseases specialization-

A predominant reason was the dynamic and evolving nature of the field, with frequent emergence of new infections and a broad spectrum of clinical presentations. Unlike certain other chronic conditions, many infectious diseases are curable, and physicians found it rewarding to witness significant clinical improvement and enhanced quality of life in patients following appropriate treatment.

“*There is always newer diseases and your patients become absolutely well once you treat them well, so. Those sort of push me into it*”. [P1]

*“It is something that is preventable and treatable, so it still gives you hope to life. So, unlike you know, there are several diseases which, wherein you can't do much and you just, you know, patients… Patients live, but then they don't, they may not live very well, post treatment. Wherein infection, if you treat right, most of the majority of them, at least people can have a good quality of life, once they come out of the disease.”* [P9]

Physicians also highlighted the high burden of tropical diseases and new learnings, which created a clinical demand and professional focus toward managing these conditions.

*“So since, ah, the demand for focused, Hospital acquired infections were high. I slowly, you know, I think it is like a demand driven, ah,... process which enabled me to take- take infectious diseases.”* [P6]

- Sub-theme 1.2: Frequently encountered diseases in clinical practice:

All the physicians (10/10) reported that infectious disease patterns varied by season and region, with weekly case numbers ranging from about 3–110—showing that while cases increased sharply during certain seasons, some illnesses were seen all the year. Dengue fever was the most common tropical illness, followed by scrub typhus (especially in southern India), leptospirosis during the monsoon, and enteric fever and chikungunya during monsoon outbreaks. Malaria remained significant in areas like Cuttack and Mangalore but was less common in cities. Tuberculosis (including extrapulmonary forms) was a frequent cause of prolonged fever, while influenza (H1N1 and other strains), melioidosis, Kyasanur Forest Disease (KFD), and other rickettsial and Salmonella infections were also reported in various regions

*“We would see dengue very commonly, scrub typhus and we would see some typhoid, chikungunya and rarely we see malaria. So, this would be the commonest acute febrile illness”* [P1]

*“Frequently we see dengue, malaria, leptospirosis, melioidosis. scrub typhus. Very occasionally Kyasanur forest disease. These are the common ones that we see”* [P8]

*“So we get about six of them. So most common being scrub typhus and leptospirosis. We also get dengue and malaria. And now we are getting more of KFD's and salmonella, sometimes if they come as a acute febrile illness.”* [P5]

*“That depends on season. Sometimes I can see 30 also (Smiling). Depending upon the season ma… (Smiling). This, uh.. today… marginally today I haven’t seen anything which is tropical.”* [P9]

*“You can say quite an outpatient wise quite a big number actually, at least 110 patients I will see in a day with either dengue or. Dengue, or influenza, like illness or acute diarrheal disease. I see at least 10 patients per day.”* [P7]

**Fig 2:**
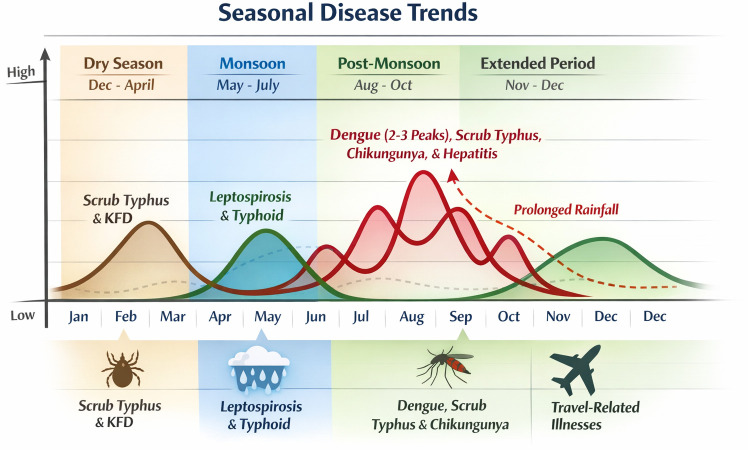
Seasonal disease trend of the disease.

- Sub-theme 1.3: Seasonal pattern of illness:

Physicians consistently reported that tropical diseases show clear seasonal trends and this has been pictorially depicted in Fig 2:

- Dengue fever and scrub typhus peak during the post-monsoon months (August–October), with dengue showing 2–3 peaks annually.
- Leptospirosis occurs mainly in the monsoon season, while typhoid and gastroenteritis rise at the end of summer due to poor water quality and hygiene.
- Mosquito-borne diseases (malaria, dengue) increase when rainfall decreases, as heavy rain suppresses breeding.
- Some physicians reported an extended disease transmission period, typically from May to December. In years with prolonged or continuous rainfall, dengue cases were observed to persist throughout the year.
- Chikungunya and viral hepatitis are common post-monsoon. Travel-related illnesses (enteric fever, malaria) often seen in travelers from northern India.
- During the dry season (December–April), scrub typhus and Kyasanur Forest Disease (KFD) rise due to tick exposure in forested areas.

*“So dengue, there is a pattern. We see dengue during about two or three spikes in a year. We see typhoid also. In sort of outbreak sort of situation that is also almost many times during couple of months, rainy season especially. Leptospirosis also after the rains and during the rainy season, so that also is seasonal. Scrub typhus is the endemic. There is actually not much seasonality noted for Scrub typhus.”* [P2]

*“Usually depends on the rain pattern and… usually at the end of May once the rain starts or now rain has come in another two weeks, if no rain we will start having dengue. Actually, May to December is the common pattern for most of them and, yeah… peak season is that… and I think 2 years back we had continuous rain throughout the year, We had throughout the year also. So it will change based on the rain pattern most of the times.”* [P10]

**Theme 2: Diagnosis and challenges:** The challenges with respect to the diagnosis of tropical fevers were discussed with the physician in the clinical setting. Overlapping clinical symptoms and reliability of initial presentations and laboratory parameters, are the major challenges for the differential diagnosis of tropical fevers in the clinics. The diagnosis might also depend on the epidemiological risk factors and other factors.

- Sub-theme 2.1: Challenges with respect to current diagnostics:

Physicians highlighted that although specific diagnostic tools developed for several tropical diseases, however, their high cost, prolonged turnaround time, and limited accessibility significantly reduce their real-time clinical value. The overlapping clinical features of tropical fevers further complicate differentiation, often forcing clinicians to depend on empirical or syndromic approaches rather than confirmed diagnoses.

Serological tests were reported to be nonspecific and delayed wit respect to positive reporting, as in some diseases antibodies appear only after several days of illness, resulting in missed opportunities for early treatment. Additionally, sensitivity and specificity issues across commonly used tests lead to false positives and negatives, lowering diagnostic reliability. Even gold-standard diagnostic methods, such as blood culture and polymerase chain reaction (PCR), are limited by issues of non-availability in certain clinical settings, inherent methodological constraints, and have prolonged turnaround times, which can delay diagnosis and have poor patient outcome.

These challenges are even more exacerbated in the overburdened healthcare systems of endemic regions which struggle to meet infectious disease diagnostic demand. Although molecular tests and rapid diagnostic tests (RDTs) have improved with respect to accessibility, they are still either too costly, unevenly distributed, or unreliable due to inconsistent quality. As there is diagnostic variability exist with changes in the stage of illness, many cases might be missed or diagnosed late (Time-dependent diagnostic utility of available assays).

Physicians also emphasized the lack of multiplex or differential diagnostic tools capable of concurrently identifying multiple infections, and also the influence of viral mutations which modify the clinical presentations and reduces the applicability of existing tests.

*“The present available diagnostics, there are certain gaps because the serologies are nonspecific. They only after a couple of days or a week, sometimes it like for example Leptospirosis serology would be positive, maybe even after a week or so, sometime diagnosis may be delayed.”* [P2]

*“Many of them don't have diagnosis in the ahh.. first week, for example, leptospirosis. Antibody become positive only after five or seven days, by which day the window for appropriate treatment is over”* [P3]

*“Same with case for even scrub typhus. All serology become positive after about a week only. But the best time to treat is in the first one week.”* [P3]

*“There are certain organisms like leptospira, where neither they will grow in the culture nor we have any PCR tests readily available. So we have to depend on the antibody test. Then we missed the diagnosis in the first week unless we have a strong suspicion.”* [P7]

*“Chikungunya were never presented with tip of nose discoloration earlier. So now it is doing it. So these viruses undergo something called shifts and drifts.”* [P9]

- Sub-theme 2.2: Challenging diseases in clinics:

Clinicians perceive several infections including leptospirosis, scrub typhus, and enteric fever as diagnostically challenging due to their non-specific signs-symptoms and delayed or unreliable test results. As per the doctors, diagnosis of leptospirosis is the most challenging since it mimics other diseases. Its conventional diagnostic test turns positive only after the first week and PCR testing is limited to mainly to tertiary centers. Scrub typhus also presents similar challenge, especially when the characteristic ‘Eschar’ is absent. The antibody tests, does not turn positive until antibodies are produced against the causative and are unreliable in the early cycle. Enteric fevers are also difficult to diagnose, as the Widal test and blood cultures lacks sensitivity and slow, especially in rural areas. Other infections including malaria, dengue, KFD, tuberculosis, and melioidosis exhibits variable degrees of diagnostic challenges. It is easy to diagnose dengue due to availability of rapid antigen tests, whereas non-falciparum malaria, extrapulmonary TB, and melioidosis are more complicated. Overall, physicians highlighted limited diagnostic infrastructure, especially in lower level of healthcare system, significantly contributing to missed or delayed diagnoses.

*“So I think scrub typhus can be, if there is no eschar can be difficult diagnose. Dengue usually has a rash. So, it's reasonably simpler. But Typhoid can be quite difficult to diagnose because it doesn't have any unique standout features”* [P1]

*“probably scrub and typhoid? That is where we struggle. Typhoid, especially thing takes culture. It takes time, and none of the other tests are kind of conclusive. Malaria. It's OK, I think. If you are using antigen, reasonably OK. But if it is non falciparum-non vivax, then it becomes a problem. Dengue, usually we don't have that kind of challenge.”* [P10]

*“Sir. Dengue and KFD are more challenging, so then leptospirosis and Scrub typhus. Scrub typhus will usually have a predominant hallmark of that eschar, which you usually find and leptospirosis we know based on the clinical profile that they look very stable, but they have organ failures and subclinical hemorrhage will be there, so you should diagnosed clinically. For dengue and KFD are difficult to diagnose clinically because there are a lot of overlapping features.”* [P5]

- Sub theme 2.3: Overlapping clinical features:

Physicians stressed that many tropical fevers share overlapping symptoms such as high-grade fever, headache, myalgia, malaise, fatigue, nausea, loss of appetite, and rashes, which makes early diagnosis more challenging. In the first week, most cases present as undifferentiated fever without organ-specific signs, complicating differentiation based on history alone. Common laboratory findings including thrombocytopenia, leukopenia, elevated liver enzymes, and renal impairment, further overlap across diseases. Although some clinical clues (e.g., eschar in scrub typhus, jaundice in leptospirosis, rash in dengue, joint pain in chikungunya, periodic fever in malaria, and hemorrhagic signs in KFD) may suggest specific infections, these features are often inconsistent and tend to appear only as the illness progresses.

Some of the clinical or laboratory clues were considered suggestive:

- Scrub typhus: altered sensorium, presence of eschar, hepatosplenomegaly
- Leptospirosis: severe myalgia, jaundice without oliguric renal failure, conjunctival hemorrhage
- Dengue: blanching erythematous rash, leukopenia, capillary leak in severe cases
- Chikungunya: small joint arthritis, discoloration of nose tip
- Malaria: periodic fever with chills and sweats
- KFD: prolonged APTT with normal PT, high CPK, hemorrhagic features

These features were not consistently present and often emerged only after disease progression or in ICU settings.

*At least many symptoms are common for almost many of the tropical diseases, including dengue, lepto, chikungunya, malaria, enteric. So all these have,um,many overlapping symptoms. [P6]*

*I think fever is common. Then thrombocytopenia, that is common. Ah… then going forward, if they are sick, they have liver and renal dysfunction. And… that is common what we see. [P10]*

*OK, fever is a common symptom for anything. In characteristics of the fever, say for example, if it is malaria, there will be high grade fever preceded by chills and triggers and fever, followed by profuse sweating. And there can be patient, can be almost normal in between the episodes of fever, or that can be fever, one episode a day or once in 2 days. Once in three days, these are the typical manifestations, so otherwise common manifestations.*[P7]

- Sub theme 2.4: Reliability of preliminary clinical indicators for early-stage diagnosis: Physicians highlighted that early diagnosis of tropical febrile illnesses depends on recognizing clinical patterns rather than single symptoms. Preliminary indicators such as fever type, rash, gastrointestinal and mucosal involvement, jaundice, conjunctival congestion, and lymphadenopathy, along with factors like travel history and illness progression, can raise diagnostic suspicion. Thrombocytopenia, leukopenia, elevated hematocrit are few basic investigations, considered helpful for screening.

However, few doctors disagreed to the above context saying that early symptoms are non specific and specific features like rashes will appear after several days of infection making the differential diagnosis difficult. Initial laboratory parameters might be pointing towards a specific disease, however they cannot be considered as confirmatory results. Additionally, many scoring systems have been developed and published in the infectious disease domain, yet their practical utility and real world applicability is limited.

*So clinical features can be the like I mentioned, mainly six of them. Fever, rash, then involvement of, you know, mucosal areas and GI symptoms jaundice, conjunctival involvement, lymphadenopathy and rhabdomyolysis.If you take these clinical parameters 7–8 of them, that can be reliable in the first week only, where the antibody will not come positive.* [P5]

*Developing a score is quite easy, but then to translate into clinical practices where the true challenge lies, yeah. [*P1*]*

- Sub theme 2.5: Epidemiological risk factors:

Occupation, travel history, and exposure to vectors are some of the epidemiological and environmental risk factors are few of the crucial components influencing the of clinical diagnosis. Specifically, during the early stages of disease cycle when laboratory confirmation are not available. Doctors usually feel confident to use this contextual clues to narrow the diagnostic pattern. They stated that, forest exposure is suggestive of KFD or tick-borne illness. The region-specific nature of these factors and predictive algorithms poses a major challenge to developing universally applicable diagnostic tools. Environmental variability, behavioral diversity, and occupation-based exposure make it difficult to create standardized diagnostic models that work across regions.

*“Epidemiological factors means the season (Ah..) the nearby, the patients, habits and where he is traveling,the travel history. Such things are very important.” [P4]*

*There are a lot of people who work at night. Those who work with call centers and international offices, right? So they work during night time. And also farmers who work in fields they are more likely to be exposed to these mosquitoes during daytime and again coming to farmers, some people who walk or work in running water or stagnant water are at risk of leptospirosis, right.* [P8]

**Theme 3: Parameter in diagnosis:**

Physicians described that diagnostic reasoning in tropical febrile illnesses is shaped by a combination of clinical, epidemiological, and laboratory parameters. In the absence of definitive early tests, clinicians rely on structured clinical judgment grounded in local disease ecology and patient presentation.

- **Subtheme 3.1:** Integrated Clinical Decision-Making

In routine practice, physicians adopt a syndromic approach to manage non-specific febrile illnesses, guided by geography, exposure, and symptom clusters. Physicians apply **integrative frameworks** (e.g., *Modified Faine’s Criteria* for leptospirosis) combining epidemiology, symptoms, and labs. Endemic patterns inform suspicion, for example, scrub typhus in dry livestock regions, KFD in forest zones, leptospirosis in waterlogged agricultural areas, and malaria in coastal regions. Exposure to cattle, forests, or stagnant water further refines diagnostic probability. Laboratory trends such as leukopenia (dengue), normal or elevated WBC (scrub typhus, leptospirosis), eosinopenia (typhoid, leptospirosis), and CRP levels help distinguish viral from bacterial etiologies. Clinicians combine these clinical and demographic cues to iteratively narrow differential diagnoses. Clinical decision making rules provided by physicians have been provided in supplementary information S5 Text.

*“For leptospirosis we have a very defined published criteria called Modified Faine’s Criteria, which includes all what you described epidemiological thing, the clinical factors, lab parameters etc, etc and which helps us to (Ahh..) understand or the possibility of leptospirosis or try to define leptospirosis using a clinical and basic lab criteria. So for leptospirosis there is something like that. Other than that, for dengue also we have. a set of standard or most common presentations. As I mentioning, headache, retro-orbital pain and leukopenia then later lymphocytosis and thrombocytopenia. So for dengue also we have a classical picture, blood picture and symptoms. For Scrub and typhoid. (Ahh..) Really, there is nothing much, very, very specific. Or very something that would actually stand out and say that this could be scrub.”* [P2]

*“So we follow based on the demographic profile. So which area they come from? Like if they come from Davanagere, Honnali or unable that area which are more dry areas, they are more likely to have scrub typhus because that is the cattle, which is very common there. ”* [P5]

- **Subtheme 3.2:** Diagnostic Testing and Emerging Alternatives

Specific microbiological tests are often limited by timing, sensitivity, and accessibility. In the early stage of illness, blood tests and PCR may not show clear results, and even culture tests do not always detect the infection reliably. Physicians emphasized the lack of affordable, standardized diagnostics outside tertiary centers. They expressed strong interest in multiplex point-of-care (POC) tools capable of detecting multiple tropical pathogens relevant to the Indian context and anticipated that AI-based platforms integrating clinical and epidemiological data could assist in early diagnostic triage, serving as supplements, not replacements, to clinical judgment.

*“Then it comes to the lab diagnosis right after the clinical syndrome and the the basic labs.Then we come on to the specific microbiological test” [P2]*

*Other methods,(Ahh..) BioFire is a method, that is available in Microbiology department in Manipal, but it does not cover all the, tropical illnesses”* [P8]

- **Subtheme 3.3:** Data Capture and Documentation Challenges

Clinicians reported inconsistencies in data recording. While electronic Health Information Systems capture laboratory variables, clinical histories and progress notes are often handwritten, hindering data retrieval. These are the handwritten records which are difficult for reading due to scribbled nature and usually paper-based documents, make it challenging to store, retrieve, and use it effectively. New symptoms occurring during hospitalization may go undocumented. They also acknowledged the subjective clinical interpretation, where clinical management depends on clinician’s speciality & perspective and mentioned it as “Rashomon effect”. Additionally, physicians first priority is patient management rather than recording the data.

*I'm working in a facility where the -the, there is HIS, that is Health information system electronic, capturing of the health information system [P6]*

*Depends in the sense the approach depends on what you are suspecting clinically. OK, to give you an example, there is something called Rashomon Effect [P7]*

- **Subtheme 3.4:** Temporal Dynamics in Diagnosis

The ‘timeline of illness’ was acknowledged as a critical parameter of diagnosis. Disease clinical presentations and test results changes with time. They mentioned that platelet counts drops after the fifth day incase of dengue; in enteric fever week-wise progression with respect to growth of causatives, and with respect to leptospirosis or scrub typhus, complications usually elevated in the second week. Choice of test mainly depends on duration of illness, with antigen or PCR-based assays preferred early and antibody-based tests later. Physicians stressed that diagnostic models, including AI tools, must incorporate this time-sensitive feature of the disease to reflect real-world clinical scenarios. [Quote 30 & 31]

*Maybe normal in dengue in the initial 2–3 days, but suddenly dropping out of five days. [P3]*

*Whether it is in the early phase of the illness or… medium term, or it is, if it is after one week again, the picture is going to change. [P10]*

**Theme 4: AI in clinical practice:** The physicians discussed the current use and awareness of AI use in clinical practice. The key aspects to the implementation strategy including acceptability, perceived usefulness, barriers, and workflow integration have also been reviewed. They also discussed regarding their expectations with respect to the development of AI tools and their applicability to the clinical context. They also highlighted the need for transparency in the result provided by the model. With respect to the validation, implementation and integration of the AI tools into the clinical practice, there's still a gap exists between the evidence and the real world practice.

- Subtheme 4.1: Current Use and Awareness of AI in Clinical Practice (Acceptability of AI tools)

Most physicians stated limited or no experience with conventional AI diagnostic tools and labelled themselves as “AI illiterate.” They articulated the need for structured training before profound adoption. A few mentioned using platforms like ChatGPT for clinical decision support system, though there was ambiguity about whether systems like “Track Care” be suitable as AI.

*“I ah… know. I mean, I don't know if my ‘track care’ is AI with that too, it is, right? Something that we see there, this thing, that, is also kind of a AI. So that's what we have used”* [P9]

*“I have not used. I'm not that tech savvy to use those things, but a few of our friends they have used. They say if you give a clinical picture to our chart ChatGPT or Meta, we get differential diagnosis that that really helps.”* [P7]

- Subtheme 4.2: Clinical Applicability and Expectations (Perceived usefulness)

Clinicians perceive AI as potentially effective for differential diagnosis of tropical fevers at all healthcare levels particularly in resource-limited and high-patient-load clinical care setting. They believed AI could aid in early diagnosis, guide test prioritization, and improve diagnostic efficiency in tertiary as well as quaternary care.

Physicians reported the overall diagnostic accuracy required to be above 85–95% and specificity-sensitivity more than 90–95%. They mentioned that current clinical expertise and serological methods already exceeds 80–90% and hence greater accuracy is anticipated than what is currently available. AI is seen as a supportive aid rather than a replacement for human judgment, with validation across centers considered to be essential.

*“AI outcome should be more specific and better than physician himself since we are using large data. And when they can only diagnose perfectly 80-90%, AI should be more than that”* [P2]

*“Yeah, see. It may be it may sound a little paradoxical. It will be most beneficial in places where there is no infrastructure to evaluate the patients in a conventional way. Because say for example in a place like Bangalore where I work. We have access to all investigations. And most of the patients are either covered with insurance or affordable to get those tests done to conclude the diagnosis. In a smaller cities, there are two cities or smaller places where there is no lab facility. Patient may or may not be affordable also. I think a lot of time that treatment is based on the clinical diagnosis rather than the laboratory conclusions. If the accuracy level, if it exceeds the clinician's predictions, accuracy level of diagnosis by a tool exceeds the clinician's diagnosis. Then I think it will make a difference.”* [P7]

- Subtheme 4.3: Model Development and Feature Selection (Design expectations)

Clinicians highlighted the necessity for comprehensive data capture across disease stages, including demographics, epidemiologic exposure, symptoms, lab results, therapy history, and outcomes.

They stressed the importance of local epidemiology and its reflection in AI, provide interpretable predictions, offer differential diagnoses, and exhibit lab trends over time. Explainability (“no black box”) was viewed as critical for clinical acceptance, with tools like SHAP or LIME cited as useful for transparency.

*“So you need to collect data regarding everyone of their symptoms, duration of symptoms, nature of those symptoms. Right. Let's say for example if you talk about fever. So in fever in the history, there are so many questions that we ask right, on set of duration, severity, you know diagonal variation. As well as you know what it responds to, what makes it worse, what time of the day all those will be there. So each of these have to be captured.”* [P8]

*“Need to open the black box”* [P3]

- Subtheme 4.4: Validation, Integration, and Implementation Challenges (Barriers and feasibility)

Physicians empathised the need for real-world validation to be qualified for trusted clinical use of AI. For the reason that, regional disease patterns, patient heterogeneity, and incomplete clinical histories the few factors that could influence the validation. They also stressed, seamless integration of such tools with electronic medical records (EMRs) and laboratory databases, which could reduce works such as manual data entry and help in improved workflow efficiency. However, this integration necessitates a strong information technology (IT) infrastructure including secure servers, and dedicated maintenance support. In addition, healthcare professionals needs continuous training to use the updated technologies.

Ethical considerations also play a critical role, including ensuring data privacy, informed consent, and appropriate anonymization. Transparent and responsible use of AI systems can enhance patient confidence, improve adherence, and increase the trust in healthcare institutions.

*“The challenge will be only when, -- once it is proven to be useful, I don't find much of a problem. Except that we need a good IT. For example, if you have to be implemented. The main challenge is to validate it.”* [P3]

*“Yeah. For example, who will enter all these variables into the.. That's the challenge. So, ah, if a particular health system, for example, ah, anywhere the labs are coming in the electronic medical record only. So the electronic, the AI has to be integrated to the current electronic-electronic medical records of the hospital. So that you know it will be an, it has to be integrated to the current electronic health record system, then only it will become useful. For example, if it is just an app, then who is going to populate this app? Nobody has time, right? So the challenges integrating the AI to the existing healthcare system”* [P6]

- Subtheme 4.5: Data Quality and Model Validation Requirements (Trust and reliability requirements)

Trust in AI tools depends on accurate data set that include clinical, epidemiologic, and lab data, used for tool development and prospective validation of the tool. Clinicians expect that, external validation needs to be ideally conducted within six hours of admission, to be the ideal decision support system and provide timely decision especially in critical care.

*“So, I would first think that, how they investigators thought to their process very carefully. And I would really think what they are allowed to enter into the algorithm. So how they set up a really good data set. First prospectively, to enter the algorithm and train the algorithm, if that is very patchy, I would not even use the algorithm.”*[P1]

*“Validation is the one challenge. Internal validation then external validation. If all that is done, then it's just question of having proper servers and um… getting ah… the… variable fed in and then business model. How we want to take it forward.”* [P3]

Overall, physicians’ perspectives across themes suggest that while AI-based tools are perceived as potentially useful for supporting differential diagnosis, their adoption in clinical practice depends on multiple factors. These include trust in model outputs, integration within existing workflows, availability of high-quality data, and context-specific applicability. Key barriers identified include limitations in diagnostic infrastructure, variability in clinical presentation, and challenges in data capture. These findings highlight the importance of aligning AI tool development with real-world clinical needs and constraints.

## Discussion

Tropical fevers characterised by acute febrile illness are the major cause of mortality and morbidity, especially in low and middle income countries. Diagnostic dilemma is the prominent issue with respect to its clinical management and mainly attributed to overlapping clinical presentations, limited access to diagnostic facilities, expensive diagnostics, and delay in availability of results. Although diseases such as dengue, and scrub typhus have conventional diagnostic tests, their clinical utility is often restricted by the issues such as delayed turnaround time, and affordability [5,10,19]. This is true especially in the resource limited settings. Overall, this study aimed to understand the perception of physicians with respect to tropical fevers and adopting the AI for differential diagnosis of tropical fevers in their clinical practice. As physicians are the end users of the diagnostic support systems, to use such tools in routine clinical practice, it is essential to encapsulate their perception during developmental phase of the models. Rather than depending on structured surveys alone, the qualitative interviews can provide us a detailed insight with respect to clinical workflow especially the pattern which the physicians follow in diagnosis of tropical fevers. This view is the necessary and critical for articulating AI tools that align with real world evidence and decision-making process. Understanding the dilemmas, challenges, and barriers that physicians face in differentially diagnose tropical fevers can aid the development of AI-based tools that are clinically relevant, with acceptable performance measures, and readily adopted by healthcare professionals [7,20].

The literature already provided a few concepts about diagnosing tropical fevers, such as the nature of the illness, difficulties caused by overlapping clinical symptoms, and the moderate accuracy of the current diagnostic tests. Physicians always acknowledged the ‘Syndromic approach’ or Syndromic nature of the diseases while making differential diagnoses for these conditions [21]. Similarly, AI is helpful in clinical practice and is being employed in numerous clinical diseases. With AI as a tool, the current study seeks to expand on this context and make it unique to the differential diagnosis of tropical fevers.

Through this study, it is possible to identify the most commonly arising challenges with respect to the tropical fevers. Due to the syndromic way of presentation with non-specific and overlapping symptoms, it is very difficult to differentiate and identify the etiological cause of disease. Physicians they mainly depend on patients’ disease presentations, specifically, pattern of signs-symptoms and the local epidemiology. However, overlapping symptoms very oftenly results in diagnostic ambiguity. Additionally, the currently available different types of diagnostic tests have inherent limitations. Few serological tests are required to be tested in the second half of the disease cycle whereas yield of culture-based methods is suboptimal. Physicians perceive that, Sensitivity and specificity of available diagnostic tests vary considerably. Furthermore, due to delayed definitive diagnosis or not established diagnostic tests physicians often directed to empirical therapy.

This kind of diagnostic uncertainty, effects not only on the clinical management of the diseases but also influence the patient behaviour and their perception with respect to the treatment seeking. This has been highlighted in a surveillance study on typhoid, [22] which suggested that socio economic factors significantly influence the patient’s perception on utilisation of hospital clinical service. These diagnostic uncertainties directly or indirectly increase the cost of patient care and leads to discontinuation of the treatments due to distrust on physicians. Even it gets more difficult for physicians, to facilitate patients understanding with respect to clinical reasoning behind investigations, empirical treatment choices, or the need for follow-up. Hence patients often shift towards self-medication which results in seeking hospital care at later stage, when symptoms are severe [22].

Additionally increased caseload of the fevers during specific season gives strain to the already limited healthcare facilities [23]. Despite the development of molecular diagnostic test and rapid diagnostic tests, their accessibility is not uniform, it might be costlier than the available tests and may not be routinely performed in all healthcare clinical setting [24]. Hence many patients are usually managed under the broad diagnosis of ‘tropical fever’ or ‘undifferentiated fever’ etc rather than specific diagnostic label. This highlights the persistent gaps in timely and accurate diagnosis of acute febrile illness. Hence experience based and syndromic approach of diagnosis predominates during the early stages of patients febrile illness [25].

During the early disease cycle, patients usually presents undifferentiated fever with overlapping and non localising symptoms. Therefore, clinicians largely depend on pattern recognition and prior clinical exposure, which is often epidemiological in nature. Based on these observations, clinicians apply syndromic grouping to guide initial diagnosis, thereby allowing clinical experience to fill the early diagnostic gap [26].

Additionally, tropical fevers are strongly influenced by seasonality and regional specificity. For example, leptospirosis is most commonly observed during the monsoon season, dengue and scrub typhus during the post-monsoon period, and Kyasanur Forest Disease requires forest exposure. Consequently, diagnostic algorithms are often viewed as region specific. From the clinician’s perspective, epidemiological risk factors serve as the primary diagnostic clues, followed by consideration of patient demographics and geographic context, with laboratory confirmation used subsequently [27]. According to medical professionals, with practice, they would be able to correctly diagnose patients by grasping the diagnostic pattern, which is challenging to identify.

Current available diagnostics may not be available in all settings, may be unaffordable, or may have limited reliability in certain situations causing diagnostic delays. In few hospitals settings Complete comprehensive examination is performed only in presence of high disease incidence. When incidence is slow test cannot be routinely performed. Hence even if the disease is present delayed or missed testing can read to diagnostic delays enhancing the reliance on empirical therapy [1,26].

Become more complicated in presence of evolving disease nature which might include microbial resistance, environmental sensitivity, characteristic of the species, time dependent clinical features etc. Disease presentation changes rapidly overtime and they may not be evident at the initial presentation. The diagnostic yield also depends day of illness with limited sensitivity in early disease cycle. While late presentations present with clear disease pattern but often associated with late stage complications. In this context, AI has emerged as a potential support system to clinical decision making the finding from the study indicate that application of AI in clinical practice possess high requirement inspite of confusion with respect to its reliability and interpretability.

Although AI based diagnostic tools presents potential usefulness in clinical decision making, physician suggested that they prefer such tools to be developed as clinical decision support system which will help in diagnosis of the fevers rather than the diagnostic itself. They believe that they are more interested in the reason for the diagnosis or the likelihood of a diagnosis by the AI rather than AI's diagnostic results.

Overall clinicians had mixed opinion regarding the development and application of AI based tools for tropical fever differential diagnosis. While some of the physician perceived AI to be useful and others expressed uncertainty. The disbelief on the AI is majorly attributed to the ‘black box’ nature of AI which limits the transparency and also the explainability [28].

Notably most of the physician were of the opinion that they're requiring training for the use of AI and they are not much aware of any existing al tools specifically for the diagnosis of tropical fevers. Few physicians also suggested that the trending technology, ‘ChatGPT’ could be useful in certain situations. However, they also acknowledged that, it may not be completely reliable to resolve the issue and it might be useful in resolving confusions of no complex cases. They were also of opinion that development of AI tool for differential diagnosis of tropical fever might aid across various levels of healthcare system which includes primary, secondary, tertiary and quaternary healthcare clinical system [29].

So this study received overall positive perception on the use of AI in healthcare especially in case of differential diagnosis of tropical fevers. The participants who were already having prayer experience in using AI were supportive suggesting that AI shoot work as assistive or supportive technology rather than replacing the physicians. The specifically mentioned that AI should be used in coordination with the human expertise, where all the AI generated results should be validated by the experts [30].

Few others were off the opinion that, if the AI model undergo transparent external validation, they would get the confidence to use the AI, based on performance during the external validation. In addition to this, few physicians also anticipated challenges during implementation. These included the need of training for the professionals, necessity of obtaining explainable AI outputs, strong technical team behind etc. Before accepting AI generated results, positions desire to understand the reasons behind the recommendation provided by the model [31,32].

Our study findings highlights that the success of AI based diagnostic technologies in Tropical Medicine not only depends on the performance of the model but also based on its alignment with existing diagnostic pattern and reason behind its recommendation. Developing the AI system as adaptive clinical decision support system has more advantages rather than making it just a static diagnostic classifier.

As suggested by Norman G et al [33], the identified themes can be anchored to established models of clinical reasoning and decision-making. Specifically, clinicians accounts of experience and routine practice reflect the development of their intuitive along with their experience-based reasoning. Diagnostic challenges highlight uncertainty arising from constrained systems and overlapping clinical presentations. The use of clinical parameters shows hybrid reasoning, where heuristics are combined with analytical rules and investigations. Finally, perceptions of AI align with augmented decision-making, where trust, explainability and workflow integration shape acceptance rather than replacement of clinical judgement. Additionally, from the AI point of view, the correspondence published in Lancet journal advised that, clinical AI physicians are crucial from the perspective of AI stewardship rather than the coding itself [34]. Even the framework provided by the National Academy of Medicine in a “The Learning Health System Series” [35] suggests many limitations of using AI system. The framework addresses about fear of replacement by physicians, data bias, impact of AI on patients and healthcare provider relations, technochauvinism (belief that technology is the best solution for any problem), trust, ethics, and fairness. Also, implementation process is complex which might need the collaboration of different group of people such as clinicians, data scientist, policy makers, patients, etc. Even though it enhances the human care, it will not be able to replace the compassion, make overload physicians to manage technology. Furthermore, larger hospitals can easily adopt these systems, unlike the hospital in resource limited setting, considering equity as concern. Our study alliance with the “framework for selection and implementation of AI in healthcare”, which is suggestive of need for external validation, involvement of patients to build the trust, a strong IT support, continuous monitoring, effect of dataset shift, interpretability, need of higher-stronger evidence for adopting in clinical settings etc. As the recommendation suggests more of limitations and considering Indian clinical setting point, we obtained a moderately different answers in our study cohort. Hence, the country like India, might need its own framework for AI system development and implementation, with their own inbuilt limitations of healthcare system considering its burden of diseases, knowledge of importance of data for development, etc.

Hence our qualitative research is first of its kind which focused on exploring the physicians perspective on barriers and challenges respect to the use of an AI in differential diagnosis of tropical fever, have not been previously studied and reported. To the best of our knowledge this study represents a first of its kind to obtain the clinicians perspective on the potential role of AI with respect to complicated diagnostic pathways. Although we achieved data saturation during the research process, the relatively small sample size and use of purposive/ snowball sampling, might limit the generalizability of the findings. However, qualitative studies, by their nature, are designed to explore the depth and complexity of participants’ experiences rather than to produce generalized findings. Although certain themes may recur across participants, the frequency with which specific responses are reported cannot be interpreted in a statistical or generalizable manner. This is because qualitative data are inherently individualized and context dependent. Consequently, the study did not attempt to quantify participants’ responses, as doing so would not align with the methodological principles of qualitative research. Moreover, present study focused on collecting the information regarding the most suitable features for proposed AI based diagnostic aiding tool and their usefulness in the clinical decision making.

## Conclusion

This study represent the first of its kind qualitative interview with physicians, to obtain the perception on the challenges, barriers and dilemmas in differential diagnosis of tropical fevers. We explored the diagnostic patterns of many South Indian doctors and acquired their in-depth insights into diagnostic dilemmas. Participants emphasized the need for development of transparent system, with assistance of strong information technology, need of training to healthcare professionals, integration to electronic health records etc. Importance of accurate data capture, need for high quality data, significance of model validation, challenges in implementation have been identified. The study added to the body of knowledge regarding challenging diseases, seasonal patterns of diseases in India, overlapping clinical features, epidemiological risk factors, reliability on initial markers etc. Hence the study contributes to preliminary insights on overall perception of physicians on clinical management of tropical fevers, highlighting key aspects such as requirement for AI model development, its applicability, trust on the developed AI model, usability, and workflow integration. Thus, aiding in informing future AI models development and evaluation of such technologies for the successful implementation of developed instruments.

## Supporting information

S1 Text SRQR checklist. Completed Standards for Reporting Qualitative Research (SRQR) checklist used for this study.
(DOC)

S2 Text Validated interview guide. Final version of the semi-structured interview guide used for conducting interviews**.**
(PDF)

S3 Text Question–analysis table. Mapping of interview questions to the corresponding analytical framework.
(DOCX)

S4 Text Themes, subthemes, and quotations. Detailed presentation of themes, subthemes, and representative participant quotations.
(DOCX)

S5 Text Clinical decision-making rules. Summary of clinical reasoning frameworks described by physicians.
(PDF)
