# Supplementary material for: A qualitative study to inform the design and implementation of AI-driven diagnosis: Challenges, barriers, and clinical insights of physicians
Source: PLoS One. 2026 May 22;21(5):e0348519. doi: 10.1371/journal.pone.0348519 (PMC13196980; doi:10.1371/journal.pone.0348519)
Supplement: S1 Text — Completed Standards for Reporting Qualitative Research (SRQR) checklist used for this study. (DOC) [file pone.0348519.s001.doc]

**Standards for Reporting Qualitative Research (SRQR)**

O’Brien B.C., Harris, I.B., Beckman, T.J., Reed, D.A., & Cook, D.A. (2014). Standards for reporting qualitative research: a synthesis of recommendations. *Academic Medicine, 89(9)*, 1245-1251.

| **No. Topic** | **Item** |
| --- | --- |
| **Title and abstract** |  |
| S1 Title | Page 1 (Title page) |
| S2 Abstract | Page 2 (Abstract section) |
| **Introduction** |  |
| S3 Problem formulation | Pages 3–5 (Introduction – background and rationale clearly described) |
| S4 Purpose or research question | Page 5 (Research objectives stated at end of introduction) |
| **Methods** |  |
| S5 Qualitative approach and research paradigm | Page 5-6 (Methodology section): Phenomenological qualitative approach with abductive reasoning; interpretivist/constructivist paradigm implied |
| S6 Researcher characteristics and reflexivity | Page 7 (Interview process): Interviews conducted by a trained PhD student; limited details on researcher reflexivity provided. |
| S7 Context | Pages 5–8: Study conducted across multiple centers in India including tertiary and other healthcare settings; includes contextual factors such as resource variability and endemic disease patterns. |
| S8 Sampling strategy | Pages 5–6: Purposive and snowball sampling used; inclusion criteria defined; sampling continued until data saturation (achieved after 8 interviews, confirmed with 10 participants). |
| S9 Ethical issues pertaining to human subjects | Page 6: Institutional Ethics Committee approval obtained (IEC number: 6/2024); informed consent obtained; confidentiality maintained through anonymization. |
| S10 Data collection methods | Pages 7–8: Semi-structured interviews conducted (face-to-face/online); audio-recorded; conducted between July 2024 and April 2025; iterative process until data saturation. |
| S11 Data collection instruments and technologies | Pages 6–8: Validated semi-structured interview guide developed using Interview Protocol Refinement (IPR) framework; pilot tested; interviews audio-recorded. And used Atlas ti for data management |
| S12 Units of study | Page 7 (Interview process): 10 physicians included; experience ranging 10–30 years; recruited from diverse clinical settings. |
| S13 Data processing | Page 8: Interviews transcribed verbatim; anonymized; transcripts reviewed for accuracy; coded using ATLAS.ti software. |
| S14 Data analysis | Pages 7–8: Reflexive thematic analysis using Braun and Clarke framework with abductive approach; coding performed by two researchers and validated by subject expert. |
| S15 Techniques to enhance trustworthiness | Pages 6–8: Multiple coders used; expert validation; iterative coding; data saturation ensured. |
| **Results/Findings** |  |
| S16 Synthesis and interpretation | Pages 8–18 (Results section): Four major themes identified—(1) clinical experience and practice, (2) diagnostic challenges, (3) diagnostic parameters and decision-making, and (4) AI integration in clinical practice; includes interpretive analysis of physicians’ perspectives. |
| S17 Links to empirical data | Pages 9–18: Participant quotes used throughout the Results section to support identified themes. |
| **Discussion** |  |
| S18 Integration with prior work, implications, transferability, and contribution(s) to the field | Pages 18–21 (Discussion): Findings compared with existing literature on diagnostic challenges and AI in healthcare; implications for clinical practice and AI implementation discussed; highlights contribution to understanding AI adoption in tropical disease diagnosis. |
| S19 Limitations | Page 21: Limitations discussed, including small sample size, and potential lack of generalizability. |
| **Other** |  |
| S20 Conflicts of interest | Page 22 (Declarations section): Authors declare no conflicts of interest. |
| S21 Funding | Not reported in manuscript |

^a^The rationale should briefly discuss the justification for choosing that theory, approach, method, or technique rather than other options available, the assumptions and limitations implicit in those choices, and how those choices influence study conclusions and transferability. As appropriate, the rationale for several items might be discussed together.
