# Supplementary material for: A qualitative study to inform the design and implementation of AI-driven diagnosis: Challenges, barriers, and clinical insights of physicians
Source: PLoS One. 2026 May 22;21(5):e0348519. doi: 10.1371/journal.pone.0348519 (PMC13196980; doi:10.1371/journal.pone.0348519)
Supplement: S2 Text — Final version of the semi-structured interview guide used for conducting interviews. (PDF) [file pone.0348519.s002.pdf]

Demographics details:

Age Group	21-30
	31-40
	41-50
	51-60
	>61
Gender:	Male ▢ Female ▢
Speciality of clinical practice:	Clinical Microbiology
	General Medicine
	Infectious Disease
	Critical Care Specialist
	Allied Health Practitioner
	Medical student
Experience level:	Intern
	Junior Physician
	Senior Physician
	Consultant/Expert
	Predominate Ward/Unit based
Any current role in addition to clinical role:	
Have you formally completed any AI/ML related learning.	

Interview questions:

Clinical experience and practice	1. How long have you been involved in infectious disease management?
	2. What are the most common tropical fevers frequently encounter in your clinical practice?
	3. How many cases of tropical fevers do you find in a week?
	4. What is the seasonal pattern of tropical fever cases in your area?
Diagnosis & challenges	1. Which tropical fevers in your opinion are linked to diagnostic dilemma?
	2. Why are these tropical fevers challenging to diagnose?
	3. Which aspect of treating tropical fever is the most challenging for you?
	4. Do you find it challenging to differentiate between various tropical fevers with similar initial symptoms?
	5. What are the challenges do you encounter in the clinical practice of diagnosis of tropical fever despite the availability of specific laboratory tests?
	6. In order to diagnose tropical fever, what are the preliminary signs and symptoms are you looking for?
	7. What are the primary methods you currently use for diagnosing tropical fevers?
	8. Do you belive that avaliable diagontic tests are accuarte in diagnosis with respect to your perspective
	9. What are the factors according to you affect the accuracy?
Parameters in diagnosis	1. In your experience, how do you think the clinical variables, epidemiological and demographical factors are useful in the diagnosis of tropical fever
	2. How do they influence your diagnostic decision-making process?
	3. As per your practice, which are the primary laboratory parameter important in diagnosis of the tropical fevers other than the available routine tests?
	4. Whether timeline is an important factor for considering clinical variables in diagnosis?
	5. Do you think we have proper documentation for capturing these variables?
AI in clinical practice	1. Have you used or encountered any AI tools for tropical fever diagnosis in your clinical practice?
	2. What specific features would you find most valuable in the development of the ML-AI tool for diagnosing tropical fevers?
	3. With your experience what is the accuracy level you expect the AI tool has to be adjusted?
	4. What challenges do you foresee in incorporating an AI tool?
	5. Do you think development of AI tool will help in the clinical setting? Which type of hospital clinical settings could be most benefited from such an AI tool and why? (Primary/Secondary/Tertiary)
	6. Do you have any additional comments and experience to share with respect to development of clinician assisted AI tool?
