# Supplementary material for: A qualitative study to inform the design and implementation of AI-driven diagnosis: Challenges, barriers, and clinical insights of physicians
Source: PLoS One. 2026 May 22;21(5):e0348519. doi: 10.1371/journal.pone.0348519 (PMC13196980; doi:10.1371/journal.pone.0348519)
Supplement: S3 Text — Mapping of interview questions to the corresponding analytical framework. (DOCX) [file pone.0348519.s003.docx]

|  | **Domain** | **Question** | **Type of Data** | **Variable/Concept** | **Analysis Method** |
| --- | --- | --- | --- | --- | --- |
|  | Clinical Experience | How long have you been involved in infectious disease management? | Categorical | Experience level | Descriptive summary |
|  | Clinical Experience | What are common tropical fevers encountered? | Open-ended | Disease frequency | Thematic coding |
|  | Clinical Experience | How many cases per week? | Numerical | Case load | Descriptive statistics |
|  | Clinical Experience | Seasonal pattern? | Open-ended | Seasonality | Thematic coding |
|  | Diagnosis & Challenges | Which fevers are diagnostically difficult? | Open-ended | Challenging diseases | Thematic coding |
|  | Diagnosis & Challenges | Why are they difficult? | Open-ended | Diagnostic barriers | Thematic coding |
|  | Diagnosis & Challenges | Challenges despite lab tests? | Open-ended | Diagnostic limitations | Thematic coding |
|  | Diagnosis & Challenges | Difficulty in differentiating diseases? | Open-ended | Symptom overlap | Thematic coding |
|  | Diagnosis & Challenges | Preliminary signs/symptoms used? | Open-ended | Early indicators | Thematic coding |
|  | Diagnosis & Challenges | Methods used for diagnosis? | Open-ended | Diagnostic approach | Thematic coding |
|  | Diagnosis & Challenges | Are tests accurate? | Opinion-based | Perceived accuracy | Thematic coding |
|  | Diagnosis & Challenges | Factors affecting accuracy? | Open-ended | Influencing factors | Thematic coding |
|  | Parameters | Role of clinical/epi/demographic factors? | Open-ended | Decision variables | Thematic coding |
|  | Parameters | Influence on decision-making? | Open-ended | Clinical reasoning | Thematic coding |
|  | Parameters | Important lab parameters? | Open-ended | Lab indicators | Thematic coding |
|  | Parameters | Importance of timeline? | Open-ended | Temporal dynamics | Thematic coding |
|  | Parameters | Documentation issues? | Open-ended | Data quality | Thematic coding |
|  | AI in Practice | Experience with AI tools? | Open-ended | Awareness | Thematic coding |
|  | AI in Practice | Desired features? | Open-ended | Feature expectations | Thematic coding |
|  | AI in Practice | Expected accuracy? | Opinion | Performance expectation | Thematic coding |
|  | AI in Practice | Implementation challenges? | Open-ended | Barriers | Thematic coding |
|  | AI in Practice | Usefulness of AI? | Opinion | Acceptance | Thematic coding |
|  | AI in Practice | Suitable settings? | Open-ended | Applicability | Thematic coding |
|  | AI in Practice | Additional comments? | Open-ended | General insights | Thematic coding |
