# Supplementary material for: A qualitative study to inform the design and implementation of AI-driven diagnosis: Challenges, barriers, and clinical insights of physicians
Source: PLoS One. 2026 May 22;21(5):e0348519. doi: 10.1371/journal.pone.0348519 (PMC13196980; doi:10.1371/journal.pone.0348519)
Supplement: S4 Text — Detailed presentation of themes, subthemes, and representative participant quotations. (DOCX) [file pone.0348519.s004.docx]

| **Theme** | **Sub theme** | **Verbatim Quotes** | **Participant** |
| --- | --- | --- | --- |
| 1. **Clinical experience and practice** | Reasons for selecting Infectious Diseases specialization | Uh. During my MD Medicine, my mentors were the ones which drove me to the critical care, because they were very good in critical care. And I had a good exposure in critical care, and infectious disease during my MD training, that drew me to the same field. And I wanted to remain as a physician. That's why I did not take a speciality of organ, and I, continue to take critical care because I can still continue to practice my medicine infectious disease. | P1 |
|  |  | That's a very common, challenging case which we see here. In India we have to worry about infectious disease, unlike Western countries where it's more of for non-communicable disease. Here you have no choice but to focus on that. | P3 |
|  |  | I thought that infection is probably one less taught subject, in our MD's also. MD medicine also. And there is, we've being in a tropical country, there's a lot of scope, as far as understanding of infectious disease goes. And… It is something that is preventable and treatable | P9 |
|  | Frequently encountered diseases in clinical practice | So we get about six of them. So most common being scrub typhus and leptospirosis. We also get dengue and malaria. And now we are getting more of KFD's and salmonella, sometimes if they come as a acute febrile illness. | P5 |
|  |  | Frequently we see dengue, malaria, leptospirosis, melioidosis. scrub typhus. Very occasionally Kyasanur forest disease. These are the common ones that we see | P4 |
|  |  | That was the time when I have seen in (Ah..) large number of falciparum malaria in my life. Then (Ah..) we had a so other malarias also Vivax malaria also then dengue fever is a common (A..) event here. Every year you will get outbreak, so we used to get lot of dengue fever patients, and enteric fever is an another speciality where we used to get so much patients. Another thing is scrub typhus which is very common in South India.So we are getting so much patients, scrub typhus also, and leptospirosis | P4 |
|  | Seasonal pattern of illness | Now it is dengue fever. Early it was malaria and the seasonal variation will be there where we see leptospirosis, usually around … August, September, October. And then other aaa.. viral infection infections like flu, which are again seasonal. | P3 |
|  |  | So dengue, there is a pattern. We see dengue during about two or three spikes in a year.We see typhoid also. (Ahh..) In sort of outbreak sort of situation that is also almost many times during couple of months, rainy season especially. Leptospirosis also after the rains and during the rainy season, so that also is seasonal. Scrub typhus is (Ahh..) is the endemic.There is actually not much seasonality noted for Scrub typhus. | P2 |
|  |  | So we usually see quite a bit of dengue and scrub typhus after the rainy season in Vellore, which is somewhere around July is when the rains would start and June, July after that August, September, October. So, this the time we would see a lot of tropical fevers predominantly dengue and scrub typhus | P1 |
|  |  | Usually it is around the monsoon season | P6 |
|  |  | You can say quite an outpatient wise quite a big number actually, at least 110 patients I will see in a day with either dengue or. Dengue, or influenza, like illness or acute diarrheal disease. I see at least 10 patients per day. | P7 |
|  |  | We see in ICU like somewhere around ah… 5 to 10 cases per day, sometimes around 35-40 per week. | P10 |

| **Theme** | **Sub theme** | **Verbatim Quotes** | **Participant** |
| --- | --- | --- | --- |
| 1. **Diagnosis and challenges** | Challenges with respect to current diagnostics | But also, if you look it, acute febrile illness, they present in a syndromic way and so they all have fevers, might not have any differentiating features | P1 |
|  |  | Here are certain gaps because the serologies are nonspecific. They (Ahh..) only after a couple of days or a week, sometimes it like for example Leptospirosis serology would be positive, maybe even after a week or so,sometime diagnosis may be delayed. blood culture pickup rate is only 50 to 60%, so about 40%, we have to treat empirically because we, the culture would not grow. | P2 |
|  |  | I don't think there's anything in particular critical, but maybe from a public health perspective that it might be little. The number of cases of,ah, infections,ah, is pretty high, as well when- when tropical fevers are concerned | P6 |
|  |  | Ahh.. challenges ah… fact is diagnostics, because not all of them can be diagnosed easily. And many of them don't have diagnosis in the ahh.. first week, for example, leptospirosis. Antibody become positive only after five or seven days, by which day the window for appropriate treatment is over | P3 |
|  | Challenging diseases in clinics | So I think scrub typhus can be, if there is no eschar can be difficult diagnose. Dengue usually has a rash. So, it's reasonably simpler. But Typhoid can be quite difficult to diagnose because it doesn't have any unique standout features | P1 |
|  |  | Most challenging disease for diagnosis probably would be leptospirosis. The reason being the symptoms are very non specific and it overlaps with all the other diseases which we spoke about. It can also present as pneumonitis. It can present as meningitis, it can present as just a lot of fever and kidney involvement, etc. | P2 |
|  |  | Maybe in the enteric fever | P6 |
|  |  | Ah… Now the challenge is again leptospira and ah… Scrub typhus- as I mentioned before, because we don't have a commonly available test in the first one week is when it's more crucial. | P3 |
|  |  | Probably scrub and typhoid? That is where we struggle. Typhoid, especially thing takes culture. It takes time, and none of the other tests are kind of conclusive. Malaria. It's OK, I think. If you are using antigen, reasonably OK. But if it is non falciparum-non vivax, then it becomes a problem. Dengue, usually we don't have that kind of challenge. | P9 |
|  |  | Most of them are almost equal, but leptospirosis will be very difficult | P4 |
|  | Overlapping clinical features | Fever , headache and myalgia | P2 |
|  |  | Fever, of course, is a common factor, and high-grade fever, chills is common to most of them. Ah… ah… In other conditions like flu, there will be respiratory symptoms.  But fevers like leptospira and typhoid fever and uhh… scrub typhus, they all look alike in the first one week. Only fever- undifferentiated fever. There are no specific symptoms referable to any particular organ. Although some of the symptoms are more common to some of them- not specific. For example, ah… significant altered sensorium will be more favor… in favor of scrub typhus, whereas in leptospirosis, severe body pain and uh… high grade fever, chills will be the common figure. So, these are all not very specific, similar overlap is there. That is the limitation of symptoms only for diagnosis | P3 |
|  |  | I think fever is common. Then thrombocytopenia, that is common. Ah… then going forward, if they are sick, they have liver and renal dysfunction. And… that is common what we see. | P10 |
|  |  | Fever. Fever is one of the most common myalgia, headache and then non specific symptoms like nausea, vomiting, loss of appetite, right. These are the typical, In some like dengue, some very few people will come with rash. Most will come, you know without any of these features like rash, specific features. And in some there could also be abdominal pain, right. When you talk about leptospirosis, many people do develop a jaundice over a period, especially after the first week and even earlier, in very severe cases. Severe myalgia and severe you know, sepsis related features like organ dysfunctions in both leptospirosis and streptococcus and few patients of melioidosis that we see also come with very sick, you know, condition. So if you have to differentiate like you know we have malaria and dengue who usually come without much complication in the early course of disease. But those with Leptospirosis, Scrub typhus and Melioidosis. They come very sick, most of the case, most of the times. | P8 |
|  | Reliability of preliminary clinical indicators for early-stage diagnosis | Most of the times they are same or similar. I, I don't think… they present with fever, even if the rash has to come will come after four or five days. So they are mostly similar. | P9 |
|  |  | I already said na, CBC would be a good screening tool, screening test | P6 |
|  |  | Yeah, I think some rashes are very helpful, absolutely. But all others are very non specific. As of now, you know, again, it's a pattern. It's not one sign or one symptom, is never going to give us a clue. It's the pattern. The patients background, their travel history, where they stay, duration of illness, progression of illness, what symptom came first, what symptom came next, right. And the severity of the illness, whether fever associated with chills or not, so multiple small, small points do help us. But I think some of the specific features, if they are present, then it's a no brainer, right. The moment a person comes with a fever and, you know, blanching rash, we know it is 99% dengue, right. But it's not always the case. We see lot of partial presentation, so that's challenging | P8 |
|  | Epidemiological risk factors | When I say patient preference, you know behavioral preferences like some people work during the daytime, some work during night time, so their exposure to disease are different like dengue, right? The mosquitoes bite during the daytime. So there are many people who don't go out during day. There are a lot of people who work at night. Those who work with call centers and international offices, right? So they work during night time. And they sleep during day and they generally go out, you know, in the rest of the day. They are probably more likely and also farmers who work in fields they are more likely to be exposed to these mosquitoes during daytime and again coming to farmers, some people who walk or work in running water or stagnant water are at risk of leptospirosis, right. So when I told preferences basically it could be occupation or it could be their preference of when they get exposed to the risk factor. | P8 |
|  |  | So, one is the, area which they belong to or the work like which area.. dry area or it's a wet area or forest, rainfall, then visit to forest, exposure of the cattle, exposure dry leaves and trekking and bite with either tick or mosquito vector. So these are the important factor which should be taken into consideration. The only clue that this could be the this disease, nothing else will be reliable. | P5 |

| **Theme** | **Sub theme** | **Verbatim Quotes** | **Participant** |
| --- | --- | --- | --- |
| 1. **Parameter in diagnosis** | Integrated Clinical Decision-Making | For leptospirosis we have a very defined published criteria called Modified Faine’s Criteria, which includes all what you described epidemiological thing, the clinical factors, lab parameters etc, etc and which helps us to (Ahh..) understand or the possibility of leptospirosis or try to define leptospirosis using a clinical and basic lab criteria. So for leptospirosis there is something like that. Other than that, for dengue also we have. a set of standard or most common presentations. As I mentioning, headache, retro-orbital pain and leukopenia then later lymphocytosis and thrombocytopenia. So for dengue also we have a classical picture, blood picture and symptoms. For Scrub and typhoid. (Ahh..) Really, there is nothing much, very, very specific. Or very something that would actually stand out and say that this could be scrub | P2 |
|  |  | So we follow based on the demographic profile. So which area they come from? Like if they come from Davanagere, Honnali or unable that area which are more dry areas, they are more likely to have scrub typhus because that is the cattle, which is very common there | P5 |
|  |  | So many of them have a typical syndromic approach. (Ahh..) There is a clustering of cases. We, there are some predefined or very typical symptoms associated with each of these. There are also some common lab parameters like the WBC counts, the differential in WBC count, the CRP values, the kind of elevation of liver function tests, or the transaminase that we see. Involvement of the kidney, involvement of the muscle, like with elevated muscle enzymes etc.So basically it is a pattern recognition.We tried to identify the pattern and based on the pattern we try to fit in in one of these. | P2 |
|  |  | whenever available, whenever the facility is available, PCR say for example chikungunya PCR or antigen or check for example COVID or influence the PCR test. And if we are suspecting the bacterial infection, we do blood culture. Or if there is urinary infection, urine culture. So culture, we take the appropriate sample for culture and wait for the bacteria to grow in the culture. And next, if these things are negative. Basically after the 3rd or 4th 5th day of the illness, we do the antibody based test. Of course, this will be aided by the clinical diagnosis, clinical features say. For example, if the if the person comes with the fever, headache, body pain when you know sore throat cough this I know it is the respiratory tract involvement flu like or influenza like illness. If someone comes with fever, body pain, back pain, headache. Lack of appetite. No symptoms. Localizing to one particular organ of the body. Then we know that it is a systemic infection in this situation, if someone comes with these kind of symptoms, we suspect dengue fever. If someone comes with. If someone comes with the multiple joint pains, fever history, suggestive of viral illness, with multiple joint pain, then we suspect chicken with a fever. And if someone comes with fever body pain, history suggests you have viral illness and comes with rashes itching all over the body. Particular parts of the body we call that as exanthematous fever. Then we evaluate based on. Clinical suspicion. There are few conditions where the exanthematous rashes are common. Say for example, measles, rubella, dengue cell infection, leptospirosis. All these producers. So depending on the clinical suspicion, we investigate. | P7 |
|  |  | Yeah. So basically any fever coming to you, you try to see if there's any kind of localization? So if there is a upper respiratory localization, then you would think of a viral upper respiratory. And if there is no particular localization then it will come under the bracket of fever without localization. And when you have a fever without localization, then you will tend to think of all the systemic, all the diseases, or all the fevers which can cause,ah, systemic manifestation or no specific localizing,ah, features.  So under that heading will come,dengue,lepto,scrub, enteric, malaria etc. So basically, fever without any localiza-localization, then I will think of all the tropical diseases, along with some kind of a epidemiological link, like with the recent history of travel to some areas like Tamil Nadu or ah, Odisha, where- where some of the diseases like dengue and malaria kind of very prominent. If that kind of history is also there then the chance of considering those differentials also will be there. But whereas, if a patient is in a native of his place, then I know,ah, from my practice that den,ah, enteric and malaria are not that common and may not be the case. And then I may just leave them and then continue to pretest the diseases. | P6 |
|  |  | So many of them have a typical syndromic approach. (Ahh..) There is a clustering of cases. We , there are some predefined or very typical symptoms associated with each of these. There are also some common lab parameters like the WBC counts, the differential in WBC count, the CRP values, the kind of elevation of liver function tests, or the transaminase that we see. Involvement of the kidney, involvement of the muscle, like with elevated muscle enzymes etc.So basically it is a pattern recognition.We tried to identify the pattern and based on the pattern we try to fit in in one of these. | P2 |
|  | Diagnostic Testing and Emerging Alternatives | Then (Ah..) usually for dengue fever will go for NS1 antigen and the CARTA testing is done here. (Ah..) But CARTA testing is not that much when the patient go for seroconversion CARTA testing may not be that much reliable ,for need, IgM, ELISA for dengue.  (Ah..) For lepto also we will go for a CARTA testing is there but it may not always show positivity. | P4 |
|  |  | Other methods,(Ahh..) BioFire is a method, that is available in Microbiology department in Manipal, but it does not cover all the, tropical illnesses. It may be helpful in Melioidosis and Enteric fever because they are bacteria, that grow in culture. But for viruses, I'm not sure and most of the early course disease, Biofire is a too expensive Test. If somebody comes in sepsis, septic shock then it is worth it. So that's one test that we use very rarely because of the cost. | P8 |
|  |  | When you say available routine tests, I assume that you are talking about blood counts, Peripherals smears, urine analysis, platelet count and chest X-ray. Beyond that, it will be specific for different groups. So if you ask me, the AI model should be able to give, at this point with the routine parameters, … history, clinical findings and basic laboratory test, with that AI should be able to tell me, what next tests are required. | P3 |
|  |  | For diagnosis, I mean PCR based- I mean few… tests were coming with various companies. But again cost and availability is concerned. | P10 |
|  |  | Maybe the inflammatory markers that can be one additional help. I mean, I, I do use them a lot. Maybe there are certain tests which can help you ruling in and out like procalcitonin in inflammatory systems. So those things can be a good ad. | P9 |
|  | Data Capture and Documentation Challenges | I personally feel firstly I think before we think of ML algorithm or something, there should be a systematic way of capture data and careful thinking through picture all the time points in which we will collect what all data. And unless that is standardized in a large cohort prospective, It is going to be enormously challenging to build algorithms because there will be a lot of noise. | P1 |
|  |  | But whether you were able to report it point of time for some,ah, some, something like research, then I doubt it. We don't have a system-systemic, way of capturing it, but of course, ah.  I am working in a facility where… I'm working in a facility where the -the,there is HIS, that is Health information system electronic,ah, capturing of the health information system. So yeah.ah. Many of the variables we can get, for example the lab parameters we can get, but then,ah, still history and all, history, progress notes, all those things we are still writing in the case- case sheet. So retrieval of the case sheet might be a bit difficult. | P6 |
|  |  | So outpatient means outpatient treatment means you will have limited history, limited examination findings and limited investigations, whereas somebody who is sick sometimes what happens is if I think that I can wait for two days, three days before evaluating, I may not ask for. A lot of tests in the first day. If the patient, if the patient is sick. For me, when the patient is sick, every day is important. Every hour is important, so I will. I will get the test as quickly as possible till I till I clinch the diagnosis. Hmm. So I can't wait for two days, three days, uh, before asking for the second set of investigations. So there there is a there is an urgency because sometimes these severe sepsis, the infections when it is severe, we call it as sepsis with the systemic inflammatory response syndrome in patients with severe sepsis. You don't have much time, you will have to investigate first. You have to treat them fast, otherwise we will lose. Frontal because once the patient goes into multiple organ dysfunction because of the infection. Then things can go really out of our control. And they say in in infectious disease with severe infections. So the first one hour of arrival to the hospital, the patient should get an appropriate dose of antibiotic. | P7 |
|  | Temporal Dynamics in Diagnosis | Yes, yes. It is. It's very important.  For example, platelet. Maybe normal in dengue in the initial 2-3 days, but suddenly dropping out of five days. That time frame is extremely important. All this clinical variables, they are time sensitive. Ah… So, Ah… the AI model should incorporate that, which day of fever, what finding was there. That, that has to be there. | P3 |
|  |  | Absolutely. So the timeline of the illness locked into the hospital is going to be very critical for planning these strategies when especially if you're considering to set up algorithms to diagnose. | P1 |
|  |  | Oh yeah, absolutely, absolutely. You know, when the fever or any symptom persistence started, what symptom came next? How that you know, the symptom severity or nature progressed over a period of time, all this matter | P8 |

| **Theme** | **Sub theme** | **Verbatim Quotes** | **Participant** |
| --- | --- | --- | --- |
| 1. **AI in clinical practice** | Current Use and Awareness of AI in Clinical Practice | I have not used. I'm not that tech to use those things, but a few of our friends they have used. They say if you give a clinical picture to our chart GPT or Meta, we get differential diagnosis that that really helps. | P7 |
|  |  | I ah… know. I mean, I don't know if my ‘track care’ is AI with that too, it is, right? Something that we see there, this thing, that, is also kind of a AI. So that's what we have used | P9 |
|  | Clinical Applicability and Expectations | Yeah, I personally feel you if you develop a tool like this, definitely there is a component of diagnostics, so that would be very useful in primary, secondary or even tertiary. But also, there is a component of who would require critical care, who would require high end care. So that will be very important for primary and secondary cares. So they could refer those patients up front early to a larger government Medical College or to a larger hospital, private hospital, wherever it might be. So that is again something where this tool would be very useful. So, all levels primary, secondary and tertiary for diagnostics and appropriate well from primary and secondary will be useful, yeah. | P1 |
|  |  | Those who have an EMR, electronic medical records requirement would be basic I think, because unless there is an electronic medical record, the data input, the sign symptomatology and the common laboratory parameters, etc, which the AI would probably use to come up with a, you know, Diagnostic idea or a suggestion. So that would require electronic medical records. So I think the hospital should have an EMR if they are thinking of using AI as a diagnostic tool. | P2 |
|  |  | Definitely ah… tertiary care centers. Correct no. Primary, secondary,… tertiary setup is always best and Medical College setup is best. Obviously, ah… we are more of quaternary care, and… we get all end point or all referral patients. Definitely it will help in that kind of setting. | P10 |
|  |  | I mean, unless it reaches an accuracy of 95 percentage, it is - it is unlikely to be of clinical-,ah, unlikely to be of clinically useful | P6 |
|  |  | Ah… As long as it is um… overall, umm… they come -- they talk in terms of overall accuracy, forgetting sensitive specificity, if the over accuracy is about say 84%, I'll be happy with that. | P3 |
|  |  | For a expert clinician, (A..) not a normalized person, a senior clinician, his clinical equipment and the routine test. And serological markers will give him at least an 80 to 90% specificity and sensitivity in diagnosing such conditions in the first two, two or three days. (Correcting voice) | P4 |
|  | Model Development and Feature Selection | To read certain conclusions or to rule out certain disease, or to cancel the certain disease. For example, when we look at the CBC first, we will look whether total count is high or low. If it is high, then to think of bacterial, low then to think of viral and if the platelet is low, tend to think of dengue. So,ah, ours is more of a simplistic view, but then in the parameters itself there are, in the total count itself, there is neutrophils, lymphocytes, monocytes, eosinophils, basophils and also there is MCV,MCH,MCHC. | P6 |
|  |  | So first of all, it should include patients at various phases of their illness. When I say various phases, something like what you mentioned, also, abot the timelines like, Somebody who comes in within first three days, somebody who comes in within first week, somebody who comes after a week. So they have different kinds of presentations, different laboratory pictures. Their microbiological diagnostics also change. | P2 |
|  |  | What specific feature ah… -- by giving the routine ah… epidemiological, that is history of -- history, epidemiological data and symptoms, and clinical findings, plus, basic investigations. With that fed in -- If possible time data also. Ah… I expect AI to tell me, small list of differential diagnosis. And then I go ahead with evaluating these differential diagnosis. | P3 |
|  | Validation, Integration, and Implementation Challenges | The most and probably the biggest would be about the validation, right? The validating the tool to influence your clinical decision because based on the output. How will a clinician gain confidence in changing his prescription or no, changing his management depending upon the diagnostic output. So for that it has to be really validated on the ground under real world | P2 |
|  |  | if a particular health system, for example,ah,anywhere the labs are coming in the electronic medical record only. So the electronic, the AI has to be integrated to the current electronic-electronic medical records of the hospital. So that you know it will be an, it has to be integrated to the current electronic health record system, then only it will become useful. | P6 |
|  |  | We are still seeing, because sometimes we will get some numbers which tell us, this could be helpful. But how much clinically they are beneficial, is the thing which we still have to learn | P5 |
|  | Data Quality and Model Validation Requirements | But yeah, I think this thing can only detect your, help you in a, what I feel as of now I don't know what all AI can do. But to my understanding as a clinician I think it can be an aiding, AID tool. But it cannot be a diagnostic tool as yet. Hain na.  So maybe once you keep looking at these trends, you can come up with a trend that you see very often in an infection and then later it can be extrapolated to making ah… score or say something like that that can help you later. So you will have to do it multiple times at multiple centers. Then you can come up with a better… accurate, accurate tool | P9 |
|  |  | So, I would first think that, how they investigators thought to their process very carefully. And I would really think what they are allowed to enter into the algorithm. So how they set up a really good data set. First prospectively, to enter the algorithm and train the algorithm, if that is very patchy, I would not even use the algorithm. | P1 |
