# Supplementary material for: A qualitative study to inform the design and implementation of AI-driven diagnosis: Challenges, barriers, and clinical insights of physicians
Source: PLoS One. 2026 May 22;21(5):e0348519. doi: 10.1371/journal.pone.0348519 (PMC13196980; doi:10.1371/journal.pone.0348519)
Supplement: S5 Text — Summary of clinical reasoning frameworks described by physicians. (PDF) [file pone.0348519.s005.pdf]

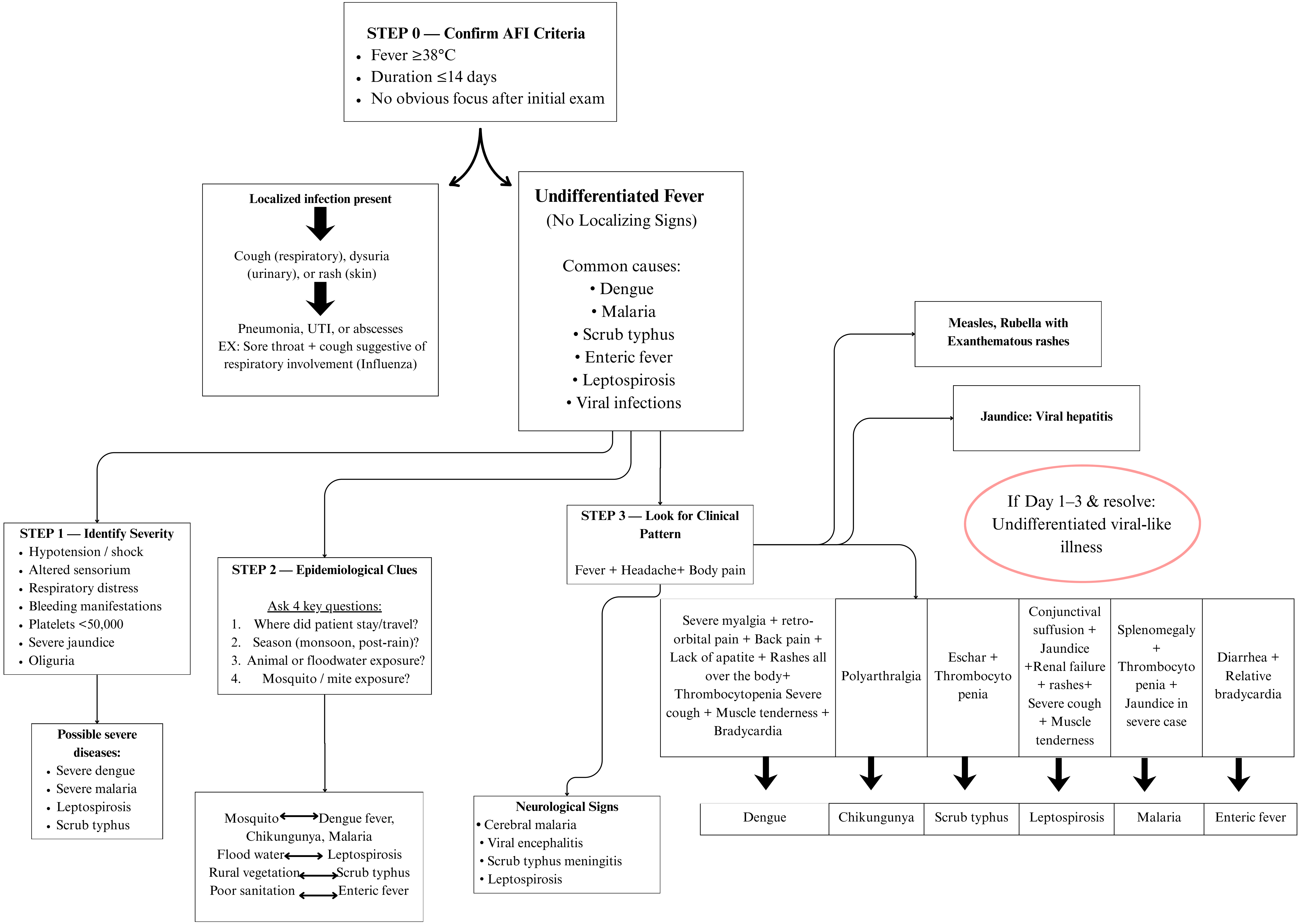
STEP 0 — Confirm AFI Criteria
• Fever ≥38°C
• Duration ≤14 days
• No obvious focus after initial exam
Localized infection present
Cough (respiratory), dysuria (urinary), or rash (skin)
Pneumonia, UTI, or abscesses
EX: Sore throat + cough suggestive of respiratory involvement (Influenza)
Undifferentiated Fever
(No Localizing Signs)
Common causes:
• Dengue
• Malaria
• Scrub typhus
• Enteric fever
• Leptospirosis
• Viral infections
Measles, Rubella with Exanthematous rashes
Jaundice: Viral hepatitis
If Day 1–3 & resolve: Undifferentiated viral-like illness
STEP 1 — Identify Severity
• Hypotension / shock
• Altered sensorium
• Respiratory distress
• Bleeding manifestations
• Platelets <50,000
• Severe jaundice
• Oliguria
Possible severe diseases:
• Severe dengue
• Severe malaria
• Leptospirosis
• Scrub typhus
STEP 2 — Epidemiological Clues
Ask 4 key questions:
1. Where did patient stay/travel?
2. Season (monsoon, post-rain)?
3. Animal or floodwater exposure?
4. Mosquito / mite exposure?
Mosquito ⟷ Dengue fever, Chikungunya, Malaria
Flood water ⟷ Leptospirosis
Rural vegetation ⟷ Scrub typhus
Poor sanitation ⟷ Enteric fever
STEP 3 — Look for Clinical Pattern
Fever + Headache+ Body pain
Neurological Signs
• Cerebral malaria
• Viral encephalitis
• Scrub typhus meningitis
• Leptospirosis
Severe myalgia + retro-orbital pain + Back pain + Lack of apatite + Rashes all over the body+ Thrombocytopenia Severe cough + Muscle tenderness + Bradycardia
Polyarthralgia
Eschar + Thrombocytopenia
Conjunctival suffusion + Jaundice +Renal failure + rashes+ Severe cough + Muscle tenderness
Splenomegaly + Thrombocytopenia + Jaundice in severe case
Diarrhea + Relative bradycardia
Dengue
Chikungunya
Scrub typhus
Leptospirosis
Malaria
Enteric fever

STEP 4 — First-Line Lab Tests

1. CBC :
- Decreased WBC count in Dengue
- Elevated WBC count in Scrub typhus/ Leptospirosis. More specific to Leptospirosis
- Normal WBC count in Scrub typhus/ Typhoid

↓

Eosinophilia in Typhoid/Leptospirosis
Neutropenia in Enteric fever

↓

Blood count
- If it is high → Bacterial
- Reduced platelet → Dengue, Enteric fever
- HB count
  - Normal → Enteric fever
  - Normal/ High → Dengue
  - Low → Malaria
- Bicytopenia [Leukopenia + thrombocytopenia] → Dengue & Chikungunya
- Reduced HB & platelet during pulmonary hemorrhage → Leptospirosis

↓

ESR & CRP → Low/Normal in Dengue
High in leptospirosis, Enteric fever, Malaria, Scrub typhus

2. Peripheral smear + malaria antigen
3. LFT & RFT → Elevated creatine + albuminuria+ Modreate elevation of LFT + Elevated Bilirubin + Creatinine Kinase + Pancreatitis → Leptospirosis

   1. AST, ALT with two to three times elevated → Leptospirosis, Enteric fever
   2. AST, ALT , will be elevated 5 to 6 times → Dengue

   Transaminitis → Scrub typhus
4. Electrolyte → Reduced K+ → Leptospirosis

5. Urine/ blood culture routine

STEP 5 — Day-of-Illness Based Testing

Day 1–5
1. Dengue NS1 antigen
2. Malaria smear
3. Blood culture (if enteric suspected)

Day 5–10
1. Dengue IgM
2. Scrub typhus IgM
3. Leptospira IgM

↓

STEP 6— Reassess Every 24–48 Hours

Monitor:
- Platelet trend
- Haematocrit
- Creatinine
- Liver enzymes

If new organ involvement → revise diagnosis.

↓

STEP 8 — If Still Undiagnosed

Consider: Viral infections, Tuberculosis, Autoimmune disease, Drug fever
